# Causes and Mechanisms Underlying Disparities and Time Trends in Alzheimer’s Disease and Related Dementias

**DOI:** 10.1093/geroni/igaa057.3120

**Published:** 2020-12-16

**Authors:** Igor Akushevich, Carl Hill, Konstantin Arbeev

## Abstract

The objective of the Symposium is to make progress in understanding the causes and mechanisms of health-related disparities in Alzheimer’s disease, related dementias and other prominent age-related diseases. Topics will cover a range of academic and administrative topics including: i) partitioning analysis of disparities and time trends in Alzheimer’s Disease and Related Dementia; ii) a structural model approach to ethnic disparities in dementia and its assessment; iii) traumatic brain injury and dementia in the Medicare population: differences in genotype and phenotype-related risk between veteran and non-veteran subsets; iv) geographic disparities in county-level prevalence of Alzheimer’s disease across the United States; and v) the role of comorbidities in the geographic disparities of AD/ADRD mortality. A focus will be made on evaluating patterns of race/ethnicity and geographic health disparities as well as changes in time trends in AD/ADRD prevalence and mortality; identifying the causes and describing the mechanisms of these respective processes, and demonstrating how they can be identified in studies using established administrative data resources such as Medicare claims databases; and demonstrating how innovative analytic approaches such as partitioning analyses, structural model approaches, and methods of latent data analyses can be used in conjunction with empirical and regression approaches to uncover previously overlooked or understudied aspects in this area of research. Analyses of such increasingly available large health datasets provides an opportunity to obtain nationally representative results based on individual-level measures that reflect the real care-related and epidemiological processes generating disparities and time trends in AD/ADRD health outcomes.

